# Adverse event signal mining and severe adverse event influencing factor analysis of Lumateperone based on FAERS database

**DOI:** 10.3389/fphar.2024.1472648

**Published:** 2024-09-23

**Authors:** Yanjing Zhang, Chunhua Zhou, Yan Liu, Yupei Hao, Jing Wang, Bingyu Song, Jing Yu

**Affiliations:** ^1^ Department of Clinical Pharmacy, The First Hospital of Hebei Medical University, Shijiazhuang, China; ^2^ The Technology Innovation Center for Artificial Intelligence in Clinical Pharmacy of Hebei Province, The First Hospital of Hebei Medical University, Shijiazhuang, China; ^3^ Department of Clinical Pharmacy, Hebei Medical University, Shijiazhuang, China

**Keywords:** lumateperone, pharmacovigilance, adverse events, FAERS, antipsychotics

## Abstract

**Background:**

Lumateperone has been approved by the Food and Drug Administration (FDA) for the treatment of schizophrenia in adults since 2019, however, there is still a lack of data report on adverse reactions in real-world settings. Conducting data mining on adverse events (AEs) associated with Lumateperone and investigating the risk factors for serious AEs can provide valuable insights for its clinical practice.

**Methods:**

AE reports in the FDA Adverse Event Reporting System (FAERS) from 2019 Q4 (FDA approval of Lumateperone) to 2024 Q1 were collected and analyzed. Disproportionality in Lumateperone-associated AEs was evaluated using the following parameters: Reporting Odds Ratio (ROR), Proportional Reporting Ratio (PRR), Bayesian Confidence Propagation Neural Network (BCPNN), and Multi-item Gamma Poisson Shrinker (MGPS). Univariate and multivariate logistic regression analyses were conducted to identify the risk factors for Lumateperone-induced severe AEs.

**Results:**

A total of 2,644 reports defined Lumateperone as the primary suspected drug was collected, including 739 reports classified as severe AEs and 1905 reports as non-severe AEs. The analysis revealed that 130 preferred terms (PTs) with significant disproportionality were based on the four algorithms, 67 (51.53%) of which were not included in the product labeling, affecting 6 systems and organs. In addition, dizziness (81 cases) was the most reported Lumateperone-associated severe AEs, and tardive dyskinesia showed the strongest signal (ROR = 186.24). Logistic regression analysis indicated that gender, bipolar II disorder, and concomitant drug use are independent risk factors for Lumateperone-associated severe AEs. Specifically, female patients had a 1.811-fold increased risk compared with male patients (OR = 1.811 [1.302, 2.519], *p* = 0.000), while patients with bipolar II disorder had a 1.695-fold increased risk compared with patients diagnosed with bipolar disorder (OR = 1.695 [1.320, 2.178], *p* = 0.000). Conversely, concomitant use of CYP3A4 inhibitors or drugs metabolized by CYP3A4 was associated with a decreased risk of severe AEs (OR = 0.524 [0.434, 0.633], *P* = 0.000).

**Conclusion:**

Collectively, this study provides critical insights into the safety profile of Lumateperone. It highlights the need for cautious use in high-risk populations, such as females and individuals with bipolar II disorder, and emphasizes the importance of monitoring for AEs, including dizziness and tardive dyskinesia. Healthcare also should remain alert to potential AEs not listed in the prescribing information to ensure medical safety.

## 1 Introduction

Schizophrenia is a common debilitating psychiatric disorder affecting approximately 1% of the global population (GBD, 2015 DALYs and HALE Collaborators, 2016). Antipsychotic therapy remains the first-line treatment for this condition (Kositsyn et al., 2023), and the first-generation antipsychotics, such as the dopamine type 2 receptor antagonist (Pilla Reddy et al., 2013) haloperidol and chlorpromazine, have been used for over half a century. While effective in alleviating positive symptoms and reducing the recurrence risk, these medications are largely ineffective against negative symptoms and cognitive impairments and may aggravate these issues due to their adverse effects (AEs) (Miyamoto et al., 2012). Second-generation antipsychotics such as clozapine and olanzapine, though generally associated with fewer extrapyramidal AEs, are linked to increased risks of weight gain and disturbance of glucose and lipid metabolism (Huhn et al., 2019). Notably, excessive blockade of dopamine receptors can also contribute to secondary negative symptoms and cognitive dysfunction (Krogmann et al., 2019).

Recent advances in neuroscience have highlighted the involvement of multiple neurotransmitter systems, including glutamatergic, serotonergic, and γ-aminobutytanergic systems (Snyder et al., 2015), in the pathophysiology of schizophrenia. This has shifted psychotropic drug development from ‘accidental discovery’ to ‘targeted synthesis’. In December 2019, Lumateperone was approved in the United States as a treatment for schizophrenia in adults (Blair, 2020). Unlike other second-generation antipsychotics, Lumateperone’s pharmacological property is characterized by a higher affinity for serotonin (5-HT2A) receptors compared with dopamine (D2) receptors, but lower affinities for α-1 and histaminergic receptors. Additionally, it functions as a presynaptic dopamine partial agonist, serotonin reuptake inhibitor, and indirect modulator of glutamatergic systems, which may contribute to a reduced side effect of weight gain and lipid metabolic abnormalities (Greenwood et al., 2021; Corponi et al., 2019).

Nevertheless, like all medications, Lumateperone is associated with potential AEs. The objective of the present study is to evaluate the safety properties of Lumateperone by analyzing the most recent data from the Food and Drug Administration (FDA) Adverse Event Reporting System (FAERS) database, which collects and analyzes adverse drug events for evaluating drug safety and efficacy (Jiang et al., 2024; Wang et al., 2024). This analysis aims to provide evidence-based guidance for its rational clinical use by evaluating severe AEs associated with the drug.

## 2 Materials and methods

### 2.1 Data source

The FAERS data were obtained quarterly and comprised seven files: demographic and administrative information (DEMO), drug information (DRUG), adverse events (REAC), patient outcomes (OUTC), report sources (RPSR), start and end dates for reported drugs (THER), and indications for use (INDI). The data were retrieved from the FAERS quarterly data extract files available at https://fis.fda.gov/extensions/FPD-QDE-FAERS/FPD-QDE-FAERS.html. Considering the self-reported nature of the database, which can include duplicates or withdrawn/deleted reports, FDA-recommended methods were used to exclude data deduplication. We extracted the PRIMARYID, CASEID, and FDA_DT fields from the DEMO table and sorted them in ascending order based on CASEID, FDA_DT, and PRIMARYID. For reports with identical CASEID, we retained the report with the largest FDA_DT value, and in cases where both CASEID and FDA_DT were identical, the report with the highest PRIMARYID value was selected. The names of AEs were encoded using the current version of the Medical Dictionary for Regulatory Activities (MedDRA 27.0) (Lu et al., 2024), from which we obtained the System Organ Class (SOC) and Preferred Terms (PT) for further analysis.

### 2.2 Data mining

In this study, the proportional imbalance method was utilized to identify signals of AEs. As illustrated in Supplementary Table S1, this method utilizes four distinct tables for analysis. Each patient was linked to a unique ‘Primary Suspect (PS) drug’ in the database, and only cases where the PS drug matches the target drug under investigation are included in the target drug population; otherwise, they are categorized into other drug populations. To enhance signal detection, we applied a combination of the Reporting Odds Ratio (ROR) (van Puijenbroek et al., 2002), Proportional Reporting Ratio (PRR) (Evans et al., 2001), Bayesian Confidence Propagation Neural Network (BCPNN) (Bate et al., 1998), and Multi-item Gamma Poisson Shrinker (MGPS) (Szarfman et al., 2002) algorithms. Each algorithm offers distinct advantages (Liu et al., 2023): ROR corrects for bias from limited reports, while PRR is less affected by missing data. BCPNN integrates data from various sources and performs cross-validation, and MGPS is particularly effective for detecting signals related to rare events. For initial screening, Preferred Terms (PTs) with reported counts of ≥3 were selected as higher values, generally indicating stronger signal strength and a more robust association between the target drug and AEs. The equations and criteria for these algorithms are shown in Supplementary Table S2.

**FIGURE 1:**
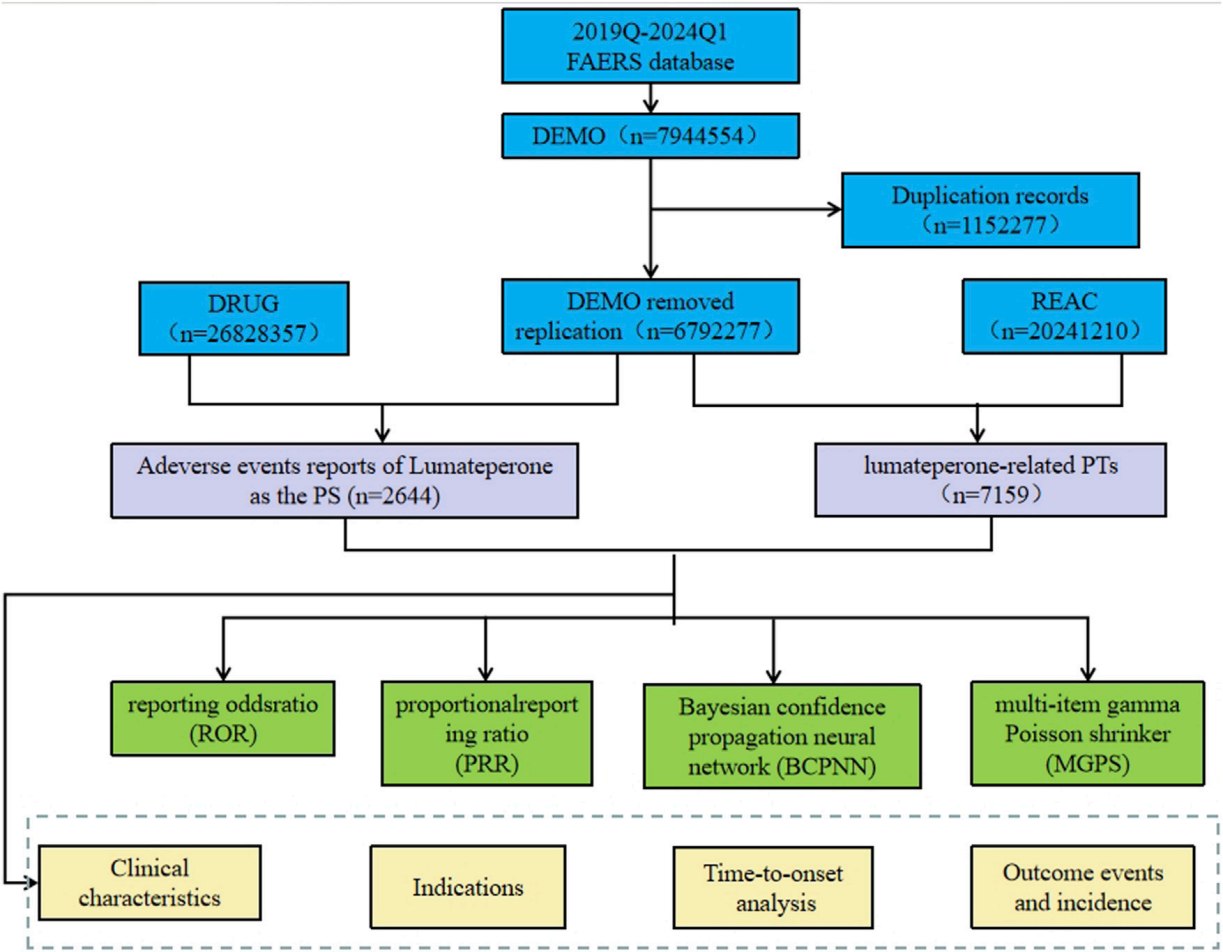
The process of extracting and analyzing Lumateperone associated adverse events from FAERS database.

Considering the timeline of drug market introduction, data from American Standard Code for Information Interchange (ASCII) report files were downloaded for the period from Q4 2019 to Q1 2024 for analysis. Comprehensive clinical characteristics of the reports were described, including gender, age, reported countries, indications, therapy start time, outcomes, reporter occupation, etc. The flowchart of data extraction, processing, and analysis is illustrated in Figure 1.

### 2.3 Factors influencing serious AEs associated with Lumateperone

AEs associated with Lumateperone were categorized into severe and non-severe AEs. Severe AEs included hospitalization (initial or prolonged), death, disability, permanent impairment or damage, life-threatening situations, congenital anomalies, or other severe medical events. Events not meeting these criteria were classified as non-severe AEs. In cases where a single report contained multiple AEs, the presence of any serious AE prompted classification of the report as severe. The frequency of AEs was presented as counts and proportions. Odds ratios (ORs) for Lumateperone-associated AEs were calculated across various exposure factors, such as gender, age, indication, and concomitant medications, using male patients aged 0–44, those diagnosed with bipolar disorder, and individuals receiving monotherapy as reference groups. Statistical analyses were performed using SPSS (v26.0; IBM Corp., Armonk, NY, United States), with statistical significance set at *p* < 0.05.

## 3 Results

### 3.1 General characteristics

From Q4 2019 to Q1 2024, a total of 7,944,554 AE reports from the FAERS database were obtained, and 2,644 reports were identified with Lumateperone as the primary suspect (PS) drug. The details were summarized in Table 1. Lumateperone-associated reports were more prevalent in female patients (58.77% vs 30.45%). The majority of patients were aged 18–64 years (41.34%). For weight categories, 2.19% of patients weighed less than 60 kg, while 9.34% weighed between 60–100 kg. The most frequently reported outcome for AEs was hospitalization (initial or prolonged) at 7.72%. Of the 299 reports with known onset time, 9.80% had an onset time within 0–30 days. Among the 999 instances of Lumateperone use with other drugs, lamotrigine was the most frequently reported concomitant drug (5.12%), followed by lithium (2.75%). The top 10 concomitant medications are listed in Table 2.

**TABLE 1:** Clinical characteristics of patients treated with Lumateperone in the FAERS database.

Characteristics	Number of events (%)
Gender	
Female (%)	1554 (58.77)
Male (%)	805 (30.45)
Not Specified (%)	285 (10.78)
Age	
<18 (%)	12 (0.45)
18–44 (%)	687 (25.98)
45–64 (%)	406 (15.36)
65–74 (%)	69 (2.61)
≥75 (%)	21 (0.79)
Not Specified (%)	1449 (54.80)
Weight(KG)	
<60 (%)	58 (2.19)
60–100 (%)	247 (9.34)
˃100 (%)	92 (3.48)
Not Specified (%)	397 (84.98)
Reporting year	
2020 (%)	126 (4.77)
2021 (%)	329 (12.44)
2022 (%)	837 (31.66)
2023 (%)	456 (17.25)
2024 (%)	896 (33.89)
Reporter	
Pharmacist (%)	1285 (48.60)
Consumer (%)	747 (28.25)
Physician (%)	598 (22.62)
Not Specified (%)	14 (0.53)
Reporting country	
United States of America (%)	2643 (99.96)
Poland (%)	1 (0.04)
Indications (top five)	
Bipolar disorder (%)	979 (37.03)
Schizophrenia (%)	386 (14.60)
Bipolar II disorder (%)	151 (5.71)
Bipolar I disorder (%)	106 (4.01)
Schizoaffective disorder (%)	50 (1.89)
Outcome	
Hospitalization - Initial or Prolonged (%)	204 (7.72)
Death (%)	28 (1.06)
Disability (%)	20 (0.76)
Required Intervention to Prevent Permanent Impairment/Damage (%)	9 (0.34)
Life-Threatening (%)	5 (0.19)
Congenital Anomaly (%)	0 (0.00)
Other (%)	529 (20.01)
Seriousness	
Non-serious	1905 (72.05)
serious	739 (27.95)
Adverse event occurrence time - medication date (days)	
0-30 d (%)	259 (9.80)
31-60 d (%)	16 (0.61)
61-90 d (%)	6 (0.23)
91-120 d (%)	3 (0.11)
121-150 d (%)	0 (0.00)
151-180 d (%)	4 (0.15)
181-360 d (%)	10 (0.38)
>360 d (%)	1 (0.04)
Unknown (%)	2345 (88.69)

**TABLE 2:** Top 10 concomitant drugs.

Concomitant medication	Reports, N (%)	Concomitant medication	Reports, N (%)
Lamotrigine	150 (5.67)	Gabapentin	65 (2.46)
Lithium	111 (4.20)	Valproic Acid	54 (2.04)
Quetiapine	86 (3.25)	Amfetamine; Dexamfetamine	51 (1.93)
Clonazepam	78 (2.95)	Hydroxyzine	47 (1.78)
Bupropion	69 (2.61)	Trazodone	45 (1.70)

### 3.2 Signal monitoring results of AEs associated with Lumateperone

A total of 2644 adverse reaction reports associated with Lumateperone were analyzed using four algorithms (ROR, PRR, BCPNN, and MGPS), resulting in the identification of 130 significant risk signals, as shown in Supplementary Table S3. Among these signals, 67 represented new AEs not previously listed on the label. Notable new AEs including psychotic disorder (n = 48, ROR = 19.59), migraine (n = 50, ROR = 4.51), feeling abnormal (n = 134, ROR = 5.55), palpitations (n = 35, ROR = 3.09), urinary incontinence (n = 17, ROR = 6.00), sexual dysfunction (n = 8, ROR = 7.37) were outlined in Table 3.

**TABLE 3:** Adverse events not documented in the label of Lumateperone at the preferred term (PT) level that fit four algorithms simultaneously.

System organ class (SOC)	Preferred terms (PT)	n	ROR (95%Cl)	PRR (chi-square)	IC (95%Cl)	IC signal strength	EBGM (95%CI)
Psychiatric disordes	Psychotic disorder	48	19.59 (14.73, 26.04)	19.46 (835.29)	4.27 (3.40, 4.23)	+++	19.34 (14.55, 25.71)
	Hallucination, auditory	43	28.11 (20.80, 37.99)	27.95 (1106.49)	4.79 (3.67, 4.55)	+++	27.68 (20.48, 37.41)
	Paranoia	38	27.73 (20.13, 38.20)	27.59 (964.52)	4.77 (3.56, 4.49)	+++	27.33 (19.84, 37.65)
	Hallucination	31	3.87 (2.72, 5.51)	3.86 (65.67)	1.95 (1.31, 2.34)	+	3.86 (2.71, 5.49)
	Aggression	30	8.07 (5.63, 11.55)	8.04 (184.46)	3.00 (2.19, 3.23)	++	8.02 (5.60, 11.48)
	Anger	25	9.06 (6.11, 13.42)	9.03 (177.96)	3.17 (2.22, 3.35)	++	9.00 (6.07, 13.34)
	Nightmare	22	7.79 (5.12, 11.84)	7.77 (129.42)	2.95 (1.98, 3.19)	++	7.75 (5.10, 11.78)
	Schizophrenia	21	13.58 (8.84, 20.87)	13.55 (242.95)	3.75 (2.49, 3.72)	++	13.49 (8.78, 20.72)
	Hypomania	16	65.12 (39.65, 106.95)	64.97 (985.22)	5.99 (3.05, 4.47)	+++	63.54 (38.69, 104.35)
	Panic attack	14	4.16 (2.46, 7.02)	4.15 (33.44)	2.05 (1.03, 2.52)	+	4.15 (2.45, 7.01)
	Delusion	13	8.24 (4.78, 14.21)	8.23 (82.29)	3.04 (1.66, 3.21)	++	8.2 (4.76, 14.15)
	Mood swings	11	4.48 (2.48, 8.11)	4.48 (29.69)	2.16 (0.96, 2.63)	+	4.47 (2.48, 8.09)
	Hallucination, visual	11	4.68 (2.59, 8.45)	4.67 (31.68)	2.22 (1.00, 2.67)	+	4.66 (2.58, 8.43)
	Euphoric mood	11	12.18 (6.73, 22.03)	12.16 (112.24)	3.6 (1.82, 3.49)	++	12.12 (6.70, 21.92)
	Fear	11	5.95 (3.29, 10.76)	5.94 (45.16)	2.57 (1.24, 2.91)	+	5.93 (3.28, 10.73)
	Abnormal behaviour	10	4.24 (2.28, 7.88)	4.23 (24.66)	2.08 (0.84, 2.58)	+	4.23 (2.27, 7.86)
	Tachyphrenia	9	30.98 (16.06, 59.79)	30.95 (257.99)	4.94 (2.03, 3.87)	++	30.62 (15.87, 59.09)
	Panic reaction	9	13.07 (6.79, 25.16)	13.05 (99.70)	3.70 (1.65, 3.48)	++	13.00 (6.75, 25.03)
	Enuresis	9	22.78 (11.82, 43.91)	22.75 (185.68)	4.50 (1.92, 3.75)	++	22.58 (11.71, 43.53)
	Catatonia	9	16.99 (8.82, 32.73)	16.97 (134.46)	4.08 (1.79, 3.62)	++	16.87 (8.76, 32.51)
	Apathy	9	6.83 (3.55, 13.14)	6.82 (44.60)	2.77 (1.19, 3.02)	+	6.81 (3.54, 13.10)
	Abnormal dreams	7	4.32 (2.06, 9.07)	4.32 (17.82)	2.11 (0.59, 2.63)	+	4.31 (2.05, 9.06)
	Bruxism	7	14.38 (6.84, 30.23)	14.37 (86.63)	3.84 (1.40, 3.45)	+	14.3 (6.80, 30.06)
	Somnambulism	7	14.58 (6.94, 30.66)	14.57 (88.02)	3.86 (1.41, 3.45)	+	14.5 (6.90, 30.48)
	Logorrhoea	7	29.34 (13.93, 61.80)	29.31 (189.45)	4.86 (1.66, 3.71)	++	29.02 (13.78, 61.13)
	Bipolar disorder	6	6.27 (2.81, 13.97)	6.26 (26.49)	2.64 (0.74, 2.93)	+	6.25 (2.81, 13.94)
	Autoscopy	6	61.72 (27.48, 138.62)	61.67 (350.45)	5.92 (1.57, 3.77)	++	60.37 (26.88, 135.60)
	Dysphemia	6	14.63 (6.56, 32.64)	14.62 (75.74)	3.86 (1.21, 3.40)	+	14.55 (6.52, 32.46)
	Distractibility	4	32.6 (12.16, 87.37)	32.58 (121.05)	5.01 (0.85, 3.45)	+	32.22 (12.02, 86.36)
	Depressive symptom	4	10.54 (3.95, 28.15)	10.54 (34.40)	3.39 (0.56, 3.15)	+	10.5 (3.93, 28.04)
	Schizoaffective disorder bipolar type	4	109.82 (40.44, 298.27)	109.76 (414.97)	6.72 (0.95, 3.59)	+	105.7 (38.92, 287.06)
	Manic symptom	4	174.03 (63.39, 477.80)	173.93 (647.88)	7.36 (0.95, 3.62)	+	163.91 (59.70, 450.01)
	Soliloquy	4	43.84 (16.33, 117.74)	43.82 (164.82)	5.43 (0.89, 3.50)	+	43.17 (16.07, 115.92)
	Emotional poverty	3	12.51 (4.02, 38.90)	12.51 (31.62)	3.64 (0.24, 3.14)	+	12.46 (4.01, 38.73)
	Intrusive thoughts	3	16.93 (5.44, 52.69)	16.92 (44.68)	4.07 (0.31, 3.21)	+	16.83 (5.41, 52.37)
	Psychotic symptom	3	9.39 (3.02, 29.19)	9.39 (22.42)	3.23 (0.15, 3.04)	+	9.36 (3.01, 29.09)
	Dissociative disorder	3	35.05 (11.22, 109.47)	35.04 (97.99)	5.11 (0.42, 3.33)	+	34.62 (11.09, 108.13)
	Trance	3	117.81 (37.11, 374.01)	117.77 (333.43)	6.82 (0.48, 3.44)	+	113.1 (35.63, 359.03)
	Homicidal ideation	3	16.31 (5.24, 50.76)	16.31 (42.86)	4.02 (0.31, 3.20)	+	16.22 (5.21, 50.46)
	Anorgasmia	3	13.34 (4.29, 41.47)	13.33 (34.06)	3.73 (0.26, 3.15)	+	13.27 (4.27, 41.28)
	Flat affect	3	22.15 (7.11, 68.99)	22.14 (60.08)	4.46 (0.36, 3.27)	+	21.97 (7.05, 68.45)
Nervous system disorders	Migraine	50	4.51 (3.41, 5.96)	4.49 (135.42)	2.16 (1.66, 2.47)	++	4.48 (3.39, 5.92)
	Paraesthesia	50	3.23 (2.45, 4.27)	3.22 (76.48)	1.68 (1.22, 2.03)	+	3.21 (2.43, 4.25)
	Disturbance in attention	33	6.29 (4.46, 8.85)	6.26 (145.70)	2.64 (1.94, 2.93)	++	6.25 (4.44, 8.80)
	Speech disorder	18	3.48 (2.19, 5.52)	3.47 (31.61)	1.79 (0.95, 2.28)	+	3.47 (2.18, 5.51)
	Electric shock sensation	15	28.63 (17.20, 47.63)	28.57 (395.09)	4.82 (2.66, 4.11)	++	28.29 (17.00, 47.08)
	Restless legs syndrome	13	6.96 (4.04, 12.00)	6.95 (66.04)	2.79 (1.51, 3.06)	++	6.93 (4.02, 11.95)
	Brain fog	10	6.74 (3.62, 12.54)	6.73 (48.71)	2.75 (1.27, 3.02)	+	6.72 (3.61, 12.50)
	Coordination abnormal	7	7.98 (3.80, 16.77)	7.98 (42.61)	2.99 (1.07, 3.11)	+	7.96 (3.79, 16.72)
	Sensory disturbance	6	4.7 (2.11, 10.47)	4.69 (17.42)	2.23 (0.53, 2.71)	+	4.69 (2.10, 10.45)
	Formication	6	11.31 (5.07, 25.23)	11.31 (56.14)	3.49 (1.10, 3.28)	+	11.26 (5.05, 25.12)
	Slow speech	5	17.18 (7.13, 41.40)	17.17 (75.69)	4.09 (1.03, 3.40)	+	17.07 (7.09, 41.14)
	Incoherent	3	6.47 (2.09, 20.11)	6.47 (13.85)	2.69 (0.00, 2.89)	+	6.46 (2.08, 20.06)
General disorders and administration site conditions	Feeling abnormal	134	5.55 (4.67, 6.58)	5.46 (488.97)	2.45 (2.15, 2.65)	++	5.45 (4.59, 6.47)
	Unevaluable event	70	8.76 (6.92, 11.09)	8.69 (475.17)	3.11 (2.62, 3.31)	++	8.66 (6.84, 10.97)
	Performance status decreased	62	169.31 (130.90, 218.98)	167.85 (9707.16)	7.31 (5.13, 5.88)	+++	158.5 (122.54, 205.00)
	Chills	41	3.27 (2.41, 4.45)	3.26 (64.18)	1.70 (1.18, 2.07)	+	3.25 (2.39, 4.42)
	Feeling drunk	22	38.38 (25.18, 58.49)	38.26 (787.80)	5.24 (3.25, 4.47)	+++	37.77 (24.78, 57.56)
	Hangover	15	47.05 (28.23, 78.41)	46.95 (663.57)	5.53 (2.87, 4.32)	++	46.2 (27.72, 77.00)
	Crying	11	3.89 (2.15, 7.02)	3.88 (23.51)	1.96 (0.81, 2.48)	+	3.88 (2.15, 7.01)
	Screaming	5	10.52 (4.37, 25.33)	10.51 (42.89)	3.39 (0.84, 3.20)	+	10.48 (4.35, 25.23)
	Energy increased	4	6.85 (2.57, 18.28)	6.85 (19.93)	2.77 (0.36, 2.95)	+	6.83 (2.56, 18.23)
Renal and urinary disorders	Urinary incontinence	17	6 (3.72, 9.65)	5.98 (70.44)	2.58 (1.54, 2.91)	++	5.97 (3.71, 9.62)
	Urinary retention	16	4.76 (2.91, 7.77)	4.75 (47.31)	2.25 (1.26, 2.66)	+	4.74 (2.90, 7.75)
	Incontinence	6	5.74 (2.58, 12.80)	5.74 (23.43)	2.52 (0.68, 2.87)	+	5.73 (2.57, 12.77)
Cardiac disorders	Palpitations	35	3.09 (2.22, 4.31)	3.08 (49.20)	1.62 (1.06, 2.02)	+	3.08 (2.21, 4.29)
Reproductive system and breast disorders	Sexual dysfunction	8	7.37 (3.68, 14.75)	7.36 (43.84)	2.88 (1.14, 3.07)	+	7.34 (3.67, 14.70)

**FIGURE 2:**
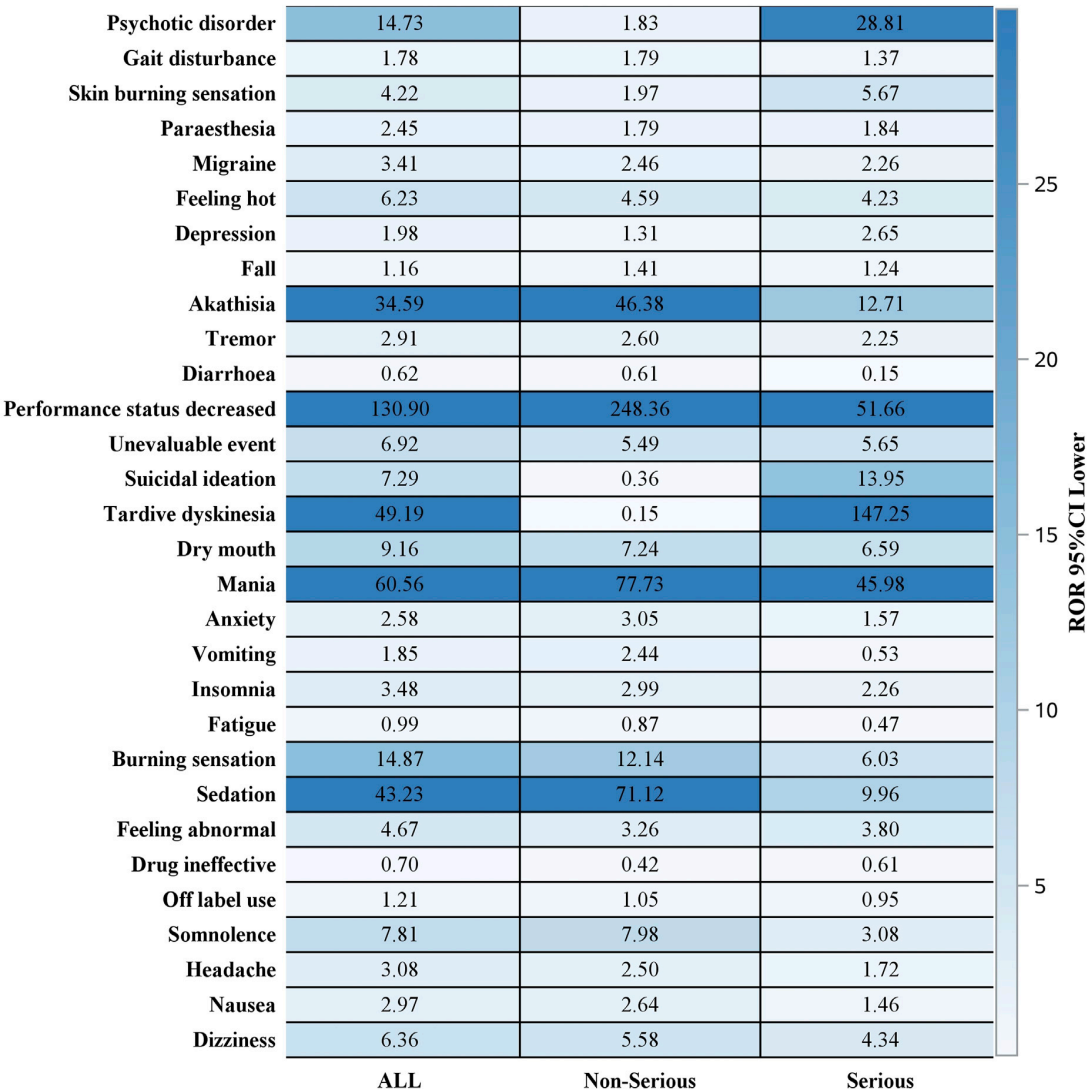
Signal strength analysis of serious vs. non-serious cases.

Out of 2644 adverse reaction reports, 739 were classified as severe. The risk signals for severe Lumateperone-related adverse reactions were ranked based on ROR strength. The top 10 validated risk signals, in descending order of ROR strength, included tardive dyskinesia, trance, schizoaffective disorder bipolar type, decreased performance status, mania, pseudostroke, hallucination, auditory, extrapyramidal disorder, neuroleptic malignant syndrome, psychotic disorder (Table 4). The occurrence risk for tardive dyskinesia and psychotic disorder was higher in the severe adverse reaction group compared to the non-severe group (Figure 2). Additionally, effective risk signals from severe reports were ranked by occurrence frequency, with the top 10 including dizziness, tardive dyskinesia, suicidal ideation, psychotic disorder, hallucination, auditory, feeling abnormal, mania, depression, somnolence and hallucination (Table 5).

**TABLE 4:** Top 10 signal strength of severe reports of Lumateperone at the Preferred terms (PTs) level in the FAERS database.

Preferred Terms	Reports	ROR (95% CI)	PRR (Chi-Square)	IC(95% CI)	EBGM(95% CI)
**Tardive dyskinesia**	**74**	**186.24 (147.25–235.56)**	**181.08 (12815.3)**	**7.45 (5.38)**	**175.11 (138.45)**
Trance	3	305.28 (95.28–978.13)	304.93 (859.25)	8.17 (0.49)	288.36 (90.00)
Schizoaffective disorder bipolar type	4	216.06 (79.44–587.62)	215.74 (821.44)	7.70 (0.97)	207.31 (76.23)
Performance status decreased	14	87.72 (51.66–148.96)	87.26 (1174.50)	6.42 (2.94)	85.86 (50.56)
**Mania**	**33**	**64.94(45.98–91.74)**	**64.15(2027.25)**	**5.99(3.98)**	**63.39(44.88)**
Pseudostroke	3	138.04 (43.84–434.60)	137.88 (397.31)	7.07 (0.50)	134.40 (42.69)
**Hallucination, auditory**	**41**	**55.27(40.54–75.37)**	**54.44(2129.29)**	**5.75(4.12)**	**53.89(39.52)**
Extrapyramidal disorder	25	55.18 (37.14–81.98)	54.67 (1303.96)	5.76 (3.58)	54.12 (36.43)
Neuroleptic malignant syndrome	22	43.99 (28.86–67.04)	43.63 (909.10)	5.44 (3.32)	43.28 (28.40)
**Psychotic disorder**	**45**	**38.73(28.81–52.05)**	**38.09(1614.16)**	**5.24(3.96)**	**37.82(28.14)**

The table is sorted by the lower 95% confidence interval of ROR, in descending order, showing only the preferred language for the positive signal. Statistical significant values are in bold.

**TABLE 5:** Top 10 severe AEs frequency of Lumateperone at the Preferred terms (PTs) level in the FAERS database.

Preferred Terms	Reports	ROR (95% CI)	PRR (Chi-Square)	IC(95% CI)	EBGM(95% CI)
Dizziness	81	5.42 (4.34–6.76)	5.29 (282.77)	2.40 (2.00)	5.28 (4.23)
**Tardive dyskinesia**	**74**	**186.24(147.25–235.56)**	**181.08(12815.3)**	**7.45(5.38)**	**175.11(138.45)**
Suicidal ideation	71	17.66 (13.95–22.37)	17.22 (1082.84)	4.10 (3.46)	17.17 (13.56)
**Psychotic disorder**	**45**	**38.73(28.81–52.05)**	**38.09(1614.16)**	**5.24(3.96)**	**37.82(28.14)**
**Hallucination, auditory**	**41**	**55.27(40.54–75.37)**	**54.44(2129.29)**	**5.75(4.12)**	**53.89(39.52)**
Feeling abnormal	34	5.32 (3.80–7.47)	5.27 (117.76)	2.40 (1.74)	5.26 (3.75)
**Mania**	**33**	**64.94(45.98–91.74)**	**64.15(2027.25)**	**5.99(3.98)**	**63.39(44.88)**
Depression	30	3.80 (2.65–5.45)	3.77 (61.18)	1.91 (1.27)	3.77 (2.63)
Somnolence	29	4.44 (3.08–6.40)	4.40 (76.32)	2.14 (1.45)	4.40 (3.05)
Hallucination	29	8.83 (6.12–12.73)	8.74 (198.74)	3.13 (2.26)	8.73 (6.05)

The table is sorted by frequency, showing only the preferred language of the positive signal. Statistical significant values are in bold.

### 3.3 Factors influencing serious AEs with lumateperone

Analysis of the correlation between patient gender, age, body weight, indication and concomitant medication with severe AEs associated with Lumateperone was presented in Table 6. Univariate and multivariate logistic regression analysis indicated that concomitant medication might influence the occurrence of severe AEs (*p* < 0.05). Our study revealed that the risk of severe AEs associated with Lumateperone was 1.811 times higher in female patients [OR = 1.811 (1.302, 2.519), *p* = 0]. Additionally, patients diagnosed with Bipolar II disorder had 1.695 times increased risk of severe AEs related to Lumateperone [OR = 1.695 (1.320, 2.178), *p* = 0]. Furthermore, the concomitant use of CYP3A4 enzyme inhibitors or drugs metabolized by CYP3A4 seem to be a protective factor against the severe adverse reactions associated with Lumateperone.

**TABLE 6:** Univariate and multivariate logistic regression analysis of the odds ratio for Lumateperone-related severe adverse events. Statistical significant values are in bold.

Variable	Factor	Univariate analysis		Multivariate analysis	
		*P*	OR (95%CI)	*P*	OR (95%CI)
Gender	Male (reference)	—	1	—	1
	Female	0	1.811 (1.302, 2.519)	0.218	1.354 (0.836, 2.193)
Age	0–44 (reference)	—	1	—	1
	45–64	0.224	0.582 (0.244, 1.391)	0.977	1.037 (0.088, 12.230)
	65–74	0.158	0.530 (0.220, 1.279)	0.887	0.835 (0.071, 9.875)
	≥75	0.417	0.665 (0.248, 1781)	0.833	0.755 (0.055, 10.322)
Body Weight	≤60	—	1	—	1
	60–100	0.537	0.813 (0.420, 1.571)	0.47	0.740 (0.327, 1.675)
	≥100	0.666	0.900 (0.557, 1.453)	0.822	0.937 (0.532, 1.651)
Indication	Bipolar disorder (reference)	—	1	—	1
	Schizophrenia	0.302	1.110 (0.910, 1.355)	0.48	0.813 (0.457, 1.445)
	Bipolar II disorder	0	1.695 (1.320, 2.178)	0.792	0.910 (0.450, 1.838)
	Bipolar I disorder	0.763	1.062 (0.720, 1.565)	0.546	1.306 (0.548, 3.112)
	Others	0.382	1.218 (0.783, 1.894)	0.89	0.934 (0.357, 2.449)
Concomitant drug	No (reference)	—	1	—	1
	CYP3A4 inhibitor or metabolized by CYP3A4	0	0.524 (0.434, 0.633)	0.569	1.175 (0.674,2.048)
	Others	0	1.797 (1.334, 2.420)	0.002	2.455 (1.407, 4.282)

## 4 Discussion

This study performed a comprehensive analysis of pharmacovigilance data regarding Lumateperone-associated AEs using post-marketing FAERS data. It offers a detailed characterization of AEs associated with Lumateperone, with a specific focus on documented instances of severe AEs, thereby enhance our understanding of their nature and prevalence.

This study found that Lumateperone-associated AE reports were more frequent in female patients compared to males (1554/58.77% and 805/30.45%, respectively). This aligns with findings by FKH et al. (Sørup et al., 2020), who identified 55 potential AEs in a psychiatric population, noting significant gender differences in frequency. These results underscore the importance of considering patient gender when evaluating the risk-benefit property of antipsychotic medications. Additionally, the frequency of AEs was higher in adults aged 18–44 years. The age of onset of schizophrenia was 20.5 years with a median age of 25 years, and this age group is likely to be at increased risk due to frequent physical comorbidities and potentially unhealthy lifestyle factors (Solmi et al., 2023).

The FAERS data predominantly reflect sources from the United States, providing insights into Lumateperone’s market within the country. The indications for Lumateperone are consistent across different countries, with no significant reports of off-label use, and the majority of reported indications align with its approved uses for bipolar disorder, schizophrenia, and other psychiatric disorders. The primary severe outcomes include hospitalization, life-threatening incidents, disability, or death. The relatively low incidence of life-threatening AEs is consistent with safety data from Lumateperone clinical trials (Jawad et al., 2022), indicating that it is relatively safe for clinical use. Our findings indicate that AEs related to Lumateperone are most commonly observed during the initial month of treatment, highlighting the necessity for heightened vigilance in monitoring patients during this period and prompt adjustments to medication methods and dosages to minimize adverse reactions. In clinical practice, antipsychotic polypharmacy, involving the combination of two or more antipsychotics, is commonly employed to address inadequate responses to antipsychotic monotherapy (Kamei, 2022). In this study, due to its mood stabilizing effect, patients with bipolar disorder or schizophrenia often used lamotrigine or lithium in combination with Lumateperone.Other drugs such as quetiapine、clonazepam, and trazodone may be associated with the comorbidities of the patients, such as depression and insomnia.

This study identified 67 new AEs related to Lumateperone through the FAERS database, involving 6 different systems and organs. These included psychiatric disorders, nervous system disorders, general disorders and administration site conditions, renal and urinary disorders, cardiac disorders, reproductive system and breast disorders. Notably, risk signals for psychiatric disorders such as psychotic disorder, hallucination, auditory and paranoia were identified, which are inconsistent with the drug label. These events not only occur frequently but also present strong signals, potentially indicative of drug-induced psychosis (Baldaçara et al., 2023). Literature reports suggest that prolonged antipsychotic therapy may exacerbate psychotic symptoms (Mishra et al., 2023). This highlights Lumateperone’s complex role in modulating multiple neurotransmitter systems and suggests a potential for exacerbating mood instability, although this issue is currently underreported, and warrants significant attention (McIntyre et al., 2023).

Risk signals associated with nervous system disorders including migraine, paraesthesia, disturbance in attention, speech disorder and electric shock sensation. The precise effects of Lumateperone on these AEs and the underlying mechanisms are not fully understood and require further clinical investigation to elucidate their potential associations and impacts.

Although drug-induced cardiac disorders associated with Lumateperone are relatively rare and currently undocumented in the literature (Correll et al., 2021), our study has revealed that Lumateperone may potentially trigger drug-induced cardiac disorders (ROR = 3.09, PRR = 3.08). Therefore, healthcare providers should consider the signs of cardiac disorders in patients undergoing treatment with Lumateperone and consider this potential association in their clinical assessments.

Analysis of severe AEs associated with Lumateperone revealed significant signals for tardive dyskinesia, psychiatric disorders, hallucinations, and mania, with some being documented in the drug instructions and others not. Therefore, it is essential to enhance patient monitoring, conduct regular laboratory testing, make timely adjustments as necessary, and ensure patient safety when administering the medication.

Our findings indicate that female patients are more likely to experience severe AEs associated with Lumateperone compared to males. This aligns with existing literature, which reports that certain side effects of antipsychotic medications, such as weight gain, passivity, low blood pressure, and hyperprolactinemia, pose particular challenges for women, with some severe side effects being more prevalent in females (Seeman, 2004). This finding is also in line with the findings of this study.

Our study indicated that the risk of severe AEs related to Lumateperone is 1.695 times higher in patients diagnosed with bipolar II disorder compared to those with bipolar disorder in general. This finding suggests that the indication of bipolar II disorder may serve as a significant risk factor for severe Lumateperone-related AEs. Bipolar II disorder, characterized by severe depressive episodes and hypomanic episodes without the full-blown manic episodes typical of Bipolar I Disorder, presents unique clinical and therapeutic challenges. Previous studies have shown that depressive episodes account for greater disability and adverse functional impact than manic or hypomanic episodes (Bobo, 2017).

Lumateperone is metabolized by various enzymes, including uridine diphosphate (UDP) glycosyltransferase (UGT)1A1, CYP3A4, CYP2C8, and CYP1A2 (Blair, 2020), resulting in over 20 metabolites. Literature indicates that co-administration of CYP3A4 inducers or inhibitors can increase the concentration of Lumateperone (Orsolini et al., 2020). In our present research, we analyzed 231 instances of Lumateperone co-administered with CYP3A4 enzyme inhibitors or drugs metabolized by CYP3A4, including quetiapine, clonazepam, trazodone, fluoxetine, and sertraline. Our analysis revealed that these concomitant medications, while influencing Lumateperone metabolism, do not independently increase the risk of severe AEs but rather appear to act as a protective factor [OR = 0.524 (0.434, 0.633), *p* = 0]. This may be due to the fact that antipsychotics metabolized by multiple CYP450 enzymes are less affected by variations in a single isoenzyme (Mulder et al., 2021). Additionally, the risk of adverse reactions is generally 2–3 times higher with combination therapy compared to monotherapy (Stassen et al., 2022), which may explain the increased risk of severe adverse reactions when Lumateperone is used with other drugs.

This study has several limitations. As Lumateperone has been approved for a relatively short period and the inherent limitations of spontaneous reporting in the FAERS database, data may be incomplete or inaccurate due to diverse global sources, potentially affecting data quality and introducing analytical biases. Most reports in our study originated from the United States, which may limit the generalizability of our findings to other regions. Additionally, the ROR, PRR, BCPNN, and MGPS methods can only identify statistical correlations between Lumateperone and AEs but cannot establish causation or elucidate the biological mechanisms underlying these associations. Therefore, while our study leverages real-world data and advanced data mining techniques, further research is needed to validate these findings and explore the underlying mechanisms (Wu et al., 2023).

## 5 Conclusion

As of 31 March 2024, we analyzed a total of 2,644 reports on Lumateperone from the FAERS database, identifying 130 significant risk signals, including 67 new AEs. These results underscore the need for healthcare providers to exercise heightened caution when prescribing Lumateperone and to inform patients about these potential AEs. Logistic regression analysis indicated that the use of concomitant medications is a risk factor for severe AEs associated with Lumateperone, while CYP3A4 enzyme inhibitors or substrates did not increase the risk of severe AEs. Despite the inherent limitations of the data, these preliminary findings provide valuable insights and reference points for future, more comprehensive studies and safety assessments.

## Data Availability

The datasets presented in this study can be found in online repositories. The names of the repository/repositories and accession number(s) can be found in the article/Supplementary Material.
