# Supplementary material for: Adverse event signal mining and severe adverse event influencing factor analysis of Lumateperone based on FAERS database
Source: Front Pharmacol. 2024 Sep 23;15:1472648. doi: 10.3389/fphar.2024.1472648 (PMC11456470; doi:10.3389/fphar.2024.1472648)
Supplement: Supplementary file 1 [file Table1.docx]

Supplementary Material

Supplementary table 1:Two-by-two contingency table for analyses.

|  | Target AE | Other AEs | Total |
| --- | --- | --- | --- |
| Lumateperone | a | b | a+b |
| All other drugs of interest | c | d | c+d |
| Total | a+c | b+d | a+b+c+d |

a=number of reports containing both the target drug and the target adverse events；

b=number of reports containing the target adverse drug reaction with other medications (except the target drug)；

c=number of reports containing the target drug with other adverse events (except the target adverse events)；

d=number of reports containing other medications and other adverse events.

Supplementary table 2. The specific formulas for the four algorithms are as follows.

| Algorithms | Equation | Criteria |
| --- | --- | --- |
| ROR | ROR=($a$/$c$)/($b$/$d$)=$\mathrm{ad}$/$\mathrm{bc}$ | a≥3 且 lower limit of 95% CI >1 |
|  | 95%CI=$e^{lnROR\pm1.96\sqrt{(\frac{1}{a}+\frac{1}{b}+\frac{1}{c}+\frac{1}{d})}}$ |  |
| PRR | $\mathrm{PRR}=\frac{a}{a+c}\div\frac{b}{b+d}$ = $\frac{a(b+d)}{(a+c)b}$  ${95\%CI=e}^{lnROR\pm1.96\sqrt{\frac{1}{a} +\frac{1}{b} + \frac{1}{c} + \frac{1}{d}}}$  χ^2^=$\frac{(ad-bc)(ad-bc)(a+b+c+d)}{(a+b)(c+d)(b+d)(a+c)}$ | PRR≥2，χ2≥4，a≥3 |
| BCPNN | IC=${log}_{2}\frac{p(x,y)}{p(x)p(y)}={log}_{2}\frac{a(a+b+c+d)}{(a+b)(a+c)}$  E(IC)=${log}_{2}\frac{(a+\gamma11)(a+b+c+d+\alpha)(a+b+c+d+\beta)}{（a+b+c+d+\gamma）(a+b+\alpha1)(a+c+\beta1)}$  V(IC)=$\frac{1}{{(ln2)}^{2}}\{\left[ \frac{\left( a+b+c+d \right)-a+\gamma-\gamma11}{\left( a+\gamma11 \right)\left( 1+a+b+c+d+\gamma\right)} \right]+\left[ \frac{\left( a+b+c+d \right)-\left( a+b \right)+\alpha-\alpha1}{\left( a+b+\alpha1 \right)\left( 1+a+b+c+d+\alpha\right)} \right]+\left[ \frac{\left( a+b+c+d \right)-\left( a+c \right)+\beta-\beta1}{\left( a+c+\beta1 \right)\left( 1+a+b+c+d+\beta\right)} \right]\}$  $\gamma=\gamma11\frac{(a+b+c+d+\alpha)(a+b+c+d+\beta)}{(a+b+\alpha1)(a+c+\beta1)}$  *IC-2SD=E(IC)-2*$\sqrt{V(IC)}$  $\alpha1=\beta1=1；\alpha=\beta=2；\gamma11=1$ | IC-2SD > 0 |
| MGPS | 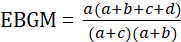 | EBGM05 > 2 |
|  | 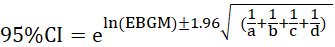 |  |

ROR, reporting odds ratio; PRR, proportional reporting ratio; BCPNN, Bayesian confidence propagation neural network; MGPS,multi-item gamma Poisson shrinker; CI，confidence interval, 95% CI, 95% confidence interval；χ^2^，chi-squared；IC，information component；IC025， the lower limit of the 95% one-sided CI of the IC；EBGM05，the lower 95% one-sided CI, of EBGM.

Supplementary table 3. 130 signifcant disproportionality PTs conforming to the four algorithms simultaneously

| System Organ Class (SOC) | Preferred terms (PT) | n | ROR(95%Cl) | PRR(Chi-Square) | IC((95%Cl)) | IC  signal  strength | EBGM(95%Cl) |
| --- | --- | --- | --- | --- | --- | --- | --- |
| Nervous system disorders | Dizziness | 335 | 7.1（6.36 ,7.92 ） | 6.81（1668.28 ） | 2.76(2.58 ,2.90 ) | ++ | 6.8(6.09 ,7.59 ) |
| Gastrointestinal disorders | Nausea | 263 | 3.35（2.97 ,3.79 ） | 3.27（418.14 ） | 1.71(1.51 ,1.88 ) | ++ | 3.27  (2.89 ,3.69 ) |
| Nervous system disorders | Headache | 225 | 3.51（3.08 ,4.01 ） | 3.43（391.13 ） | 1.78(1.57 ,1.96 ) | ++ | 3.43  (3.00 ,3.92 ) |
| Nervous system disorders | Somnolence | 181 | 9.05（7.81 ,10.49 ） | 8.85（1259.33 ） | 3.14(2.86 ,3.30 ) | ++ | 8.82(7.61 ,10.23 ) |
| General disorders and administration site conditions | Feeling abnormal | 134 | 5.55（4.67 ,6.58 ） | 5.46（488.97 ） | 2.45(2.15 ,2.65 ) | ++ | 5.45(4.59 ,6.47 ) |
| Nervous system disorders | Sedation | 126 | 51.64（43.23 ,61.68 ） | 50.74（6038.03 ） | 5.64(4.91 ,5.43 ) | +++ | 49.87(41.75 ,59.57 ) |
| Nervous system disorders | Burning sensation | 117 | 17.86（14.87 ,21.46 ） | 17.59（1820.92 ） | 4.13(3.67 ,4.21 ) | +++ | 17.49(14.56 ,21.00 ) |
| Psychiatric disorders | Insomnia | 105 | 4.22（3.48 ,5.11 ） | 4.17（253.48 ） | 2.06(1.73 ,2.30 ) | ++ | 4.16(3.43 ,5.05 ) |
| Psychiatric disorders | Anxiety | 97 | 3.15（2.58 ,3.85 ） | 3.12（140.19 ） | 1.64(1.32 ,1.90 ) | + | 3.12(2.55 ,3.81 ) |
| Psychiatric disorders | Mania | 92 | 74.59（60.56 ,91.86 ） | 73.64（6425.99 ） | 6.17(5.04 ,5.65 ) | +++ | 71.8(58.30 ,88.42 ) |
| Gastrointestinal disorders | Dry mouth | 87 | 11.33（9.16 ,14.00 ） | 11.2（805.90 ） | 3.48(3.01 ,3.63 ) | +++ | 11.16(9.03 ,13.79 ) |
| Nervous system disorders | Tardive dyskinesia | 75 | 61.9（49.19 ,77.91 ） | 61.27（4352.56 ） | 5.91(4.74 ,5.41 ) | +++ | 59.99(47.66 ,75.50 ) |
| Psychiatric disorders | Suicidal ideation | 72 | 9.19（7.29 ,11.60 ） | 9.11（518.79 ） | 3.18(2.69 ,3.37 ) | ++ | 9.08(7.20 ,11.46 ) |
| General disorders and administration site conditions | Unevaluable event | 70 | 8.76（6.92 ,11.09 ） | 8.69（475.17 ） | 3.11(2.62 ,3.31 ) | ++ | 8.66(6.84 ,10.97 ) |
| General disorders and administration site conditions | Performance status decreased | 62 | 169.31（130.90 ,218.98 ） | 167.85（9707.16 ） | 7.31(5.13 ,5.88 ) | +++ | 158.5(122.54 ,205.00 ) |
| Nervous system disorders | Tremor | 58 | 3.77（2.91 ,4.88 ） | 3.75（116.99 ） | 1.9(1.46 ,2.22 ) | + | 3.74(2.89 ,4.85 ) |
| Nervous system disorders | Akathisia | 56 | 45.08（34.59 ,58.76 ） | 44.74（2357.60 ） | 5.46(4.26 ,5.04 ) | +++ | 44.05(33.80 ,57.42 ) |
| General disorders and administration site conditions | Feeling hot | 52 | 8.19（6.23 ,10.76 ） | 8.14（324.87 ） | 3.02(2.44 ,3.24 ) | ++ | 8.12(6.18 ,10.67 ) |
| Nervous system disorders | Migraine | 50 | 4.51（3.41 ,5.96 ） | 4.49（135.42 ） | 2.16(1.66 ,2.47 ) | ++ | 4.48(3.39 ,5.92 ) |
| Nervous system disorders | Paraesthesia | 50 | 3.23（2.45 ,4.27 ） | 3.22（76.48 ） | 1.68(1.22 ,2.03 ) | + | 3.21(2.43 ,4.25 ) |
| Skin and subcutaneous tissue disorders | Skin burning sensation | 49 | 5.59（4.22 ,7.41 ） | 5.56（183.14 ） | 2.47(1.94 ,2.76 ) | ++ | 5.55(4.19 ,7.35 ) |
| Psychiatric disorders | Psychotic disorder | 48 | 19.59（14.73 ,26.04 ） | 19.46（835.29 ） | 4.27(3.40 ,4.23 ) | +++ | 19.34(14.55 ,25.71 ) |
| Psychiatric disorders | Hallucination, auditory | 43 | 28.11（20.80 ,37.99 ） | 27.95（1106.49 ） | 4.79(3.67 ,4.55 ) | +++ | 27.68  (20.48 ,37.41 ) |
| Psychiatric disorders | Agitation | 43 | 7.43（5.50 ,10.03 ） | 7.39（237.21 ） | 2.88(2.25 ,3.12 ) | ++ | 7.37(5.46 ,9.96 ) |
| Nervous system disorders | Hypersomnia | 43 | 13.97（10.34 ,18.87 ） | 13.89（512.12 ） | 3.79(2.98 ,3.86 ) | ++ | 13.83  (10.24 ,18.68 ) |
| General disorders and administration site conditions | Chills | 41 | 3.27（2.41 ,4.45 ） | 3.26（64.18 ） | 1.7(1.18 ,2.07 ) | + | 3.25(2.39 ,4.42 ) |
| Nervous system disorders | Balance disorder | 41 | 4.72（3.47 ,6.41 ） | 4.69（119.17 ） | 2.23(1.66 ,2.55 ) | ++ | 4.69(3.45 ,6.37 ) |
| Psychiatric disorders | Paranoia | 38 | 27.73（20.13 ,38.20 ） | 27.59（964.52 ） | 4.77(3.56 ,4.49 ) | +++ | 27.33  (19.84 ,37.65 ) |
| Cardiac disorders | Palpitations | 35 | 3.09（2.22 ,4.31 ） | 3.08（49.20 ） | 1.62(1.06 ,2.02 ) | + | 3.08(2.21 ,4.29 ) |
| Psychiatric disorders | Irritability | 34 | 7.51（5.36 ,10.52 ） | 7.48（190.43 ） | 2.9(2.17 ,3.14 ) | ++ | 7.46(5.32 ,10.46 ) |
| Nervous system disorders | Disturbance in attention | 33 | 6.29（4.46 ,8.85 ） | 6.26（145.70 ） | 2.64(1.94 ,2.93 ) | ++ | 6.25(4.44 ,8.80 ) |
| Nervous system disorders | Dyskinesia | 32 | 8.29（5.86 ,11.74 ） | 8.26（203.75 ） | 3.04(2.25 ,3.26 ) | ++ | 8.24(5.82 ,11.67 ) |
| Psychiatric disorders | Hallucination | 31 | 3.87（2.72 ,5.51 ） | 3.86（65.67 ） | 1.95(1.31 ,2.34 ) | + | 3.86(2.71 ,5.49 ) |
| General disorders and administration site conditions | Feeling cold | 30 | 10.53（7.35 ,15.08 ） | 10.49（256.64 ） | 3.39(2.48 ,3.52 ) | ++ | 10.45(7.30 ,14.97 ) |
| Psychiatric disorders | Aggression | 30 | 8.07（5.63 ,11.55 ） | 8.04（184.46 ） | 3(2.19 ,3.23 ) | ++ | 8.02(5.60 ,11.48 ) |
| Psychiatric disorders | Restlessness | 28 | 8.1（5.59 ,11.75 ） | 8.07（173.10 ） | 3.01(2.16 ,3.23 ) | ++ | 8.05(5.55 ,11.68 ) |
| Nervous system disorders | Extrapyramidal disorder | 27 | 27.66（18.92 ,40.44 ） | 27.56（684.51 ） | 4.77(3.27 ,4.36 ) | +++ | 27.3(18.68 ,39.91 ) |
| Psychiatric disorders | Anger | 25 | 9.06（6.11 ,13.42 ） | 9.03（177.96 ） | 3.17(2.22 ,3.35 ) | ++ | 9(6.07 ,13.34 ) |
| Nervous system disorders | Dysarthria | 22 | 6.51（4.28 ,9.90 ） | 6.5（102.14 ） | 2.7(1.78 ,2.99 ) | ++ | 6.48(4.27 ,9.86 ) |
| Psychiatric disorders | Nightmare | 22 | 7.79（5.12 ,11.84 ） | 7.77（129.42 ） | 2.95(1.98 ,3.19 ) | ++ | 7.75(5.10 ,11.78 ) |
| Nervous system disorders | Neuroleptic malignant syndrome | 22 | 23.36（15.34 ,35.56 ） | 23.29（465.52 ） | 4.53(2.95 ,4.16 ) | ++ | 23.11(15.18 ,35.18 ) |
| General disorders and administration site conditions | Feeling drunk | 22 | 38.38（25.18 ,58.49 ） | 38.26（787.80 ） | 5.24(3.25 ,4.47 ) | +++ | 37.77(24.78 ,57.56 ) |
| Psychiatric disorders | Schizophrenia | 21 | 13.58（8.84 ,20.87 ） | 13.55（242.95 ） | 3.75(2.49 ,3.72 ) | ++ | 13.49(8.78 ,20.72 ) |
| Nervous system disorders | Lethargy | 21 | 4.08（2.66 ,6.26 ） | 4.07（48.59 ） | 2.02(1.22 ,2.45 ) | + | 4.07(2.65 ,6.24 ) |
| Musculoskeletal and connective tissue disorders | Muscle twitching | 21 | 9.73（6.34 ,14.94 ） | 9.7（163.45 ） | 3.27(2.18 ,3.41 ) | ++ | 9.67(6.30 ,14.86 ) |
| Nervous system disorders | Dystonia | 20 | 15.6（10.05 ,24.23 ） | 15.56（271.12 ） | 3.95(2.56 ,3.83 ) | ++ | 15.48(9.97 ,24.04 ) |
| General disorders and administration site conditions | Temperature regulation disorder | 19 | 68.24（43.27 ,107.63 ） | 68.06（1225.99 ） | 6.05(3.30 ,4.61 ) | +++ | 66.49  (42.16 ,104.86 ) |
| Nervous system disorders | Speech disorder | 18 | 3.48（2.19 ,5.52 ） | 3.47（31.61 ） | 1.79(0.95 ,2.28 ) | + | 3.47(2.18 ,5.51 ) |
| Nervous system disorders | Cognitive disorder | 18 | 3.48（2.19 ,5.52 ） | 3.47（31.65 ） | 1.79(0.95 ,2.28 ) | + | 3.47(2.18 ,5.51 ) |
| Psychiatric disorders | Middle insomnia | 17 | 9.12（5.66 ,14.69 ） | 9.1（122.27 ） | 3.18(1.97 ,3.33 ) | ++ | 9.08(5.64 ,14.62 ) |
| Renal and urinary disorders | Urinary incontinence | 17 | 6（3.72 ,9.65 ） | 5.98（70.44 ） | 2.58(1.54 ,2.91 ) | ++ | 5.97(3.71 ,9.62 ) |
| Psychiatric disorders | Hypomania | 16 | 65.12（39.65 ,106.95 ） | 64.97（985.22 ） | 5.99(3.05 ,4.47 ) | +++ | 63.54(38.69 ,104.35 ) |
| Renal and urinary disorders | Urinary retention | 16 | 4.76（2.91 ,7.77 ） | 4.75（47.31 ） | 2.25(1.26 ,2.66 ) | + | 4.74(2.90 ,7.75 ) |
| General disorders and administration site conditions | Hangover | 15 | 47.05（28.23 ,78.41 ） | 46.95（663.57 ） | 5.53(2.87 ,4.32 ) | ++ | 46.2(27.72 ,77.00 ) |
| Nervous system disorders | Electric shock sensation | 15 | 28.63（17.20 ,47.63 ） | 28.57（395.09 ） | 4.82(2.66 ,4.11 ) | ++ | 28.29(17.00 ,47.08 ) |
| Psychiatric disorders | Disorientation | 15 | 4.65（2.80 ,7.73 ） | 4.65（42.88 ） | 2.21(1.20 ,2.64 ) | + | 4.64(2.80 ,7.70 ) |
| Psychiatric disorders | Thinking abnormal | 14 | 8.82（5.22 ,14.91 ） | 8.8（96.58 ） | 3.13(1.78 ,3.28 ) | ++ | 8.78(5.19 ,14.85 ) |
| Psychiatric disorders | Panic attack | 14 | 4.16（2.46 ,7.02 ） | 4.15（33.44 ） | 2.05(1.03 ,2.52 ) | + | 4.15(2.45 ,7.01 ) |
| Vascular disorders | Orthostatic hypotension | 14 | 7.56（4.47 ,12.77 ） | 7.54（79.26 ） | 2.91(1.64 ,3.14 ) | ++ | 7.53(4.45 ,12.72 ) |
| Musculoskeletal and connective tissue disorders | Muscle tightness | 13 | 8.64（5.01 ,14.89 ） | 8.62（87.36 ） | 3.1(1.71 ,3.25 ) | ++ | 8.6(4.99 ,14.83 ) |
| Psychiatric disorders | Delusion | 13 | 8.24（4.78 ,14.21 ） | 8.23（82.29 ） | 3.04(1.66 ,3.21 ) | ++ | 8.2(4.76 ,14.15 ) |
| Nervous system disorders | Restless legs syndrome | 13 | 6.96（4.04 ,12.00 ） | 6.95（66.04 ） | 2.79(1.51 ,3.06 ) | ++ | 6.93(4.02 ,11.95 ) |
| Nervous system disorders | Serotonin syndrome | 12 | 5.94（3.37 ,10.48 ） | 5.93（49.15 ） | 2.57(1.30 ,2.90 ) | + | 5.92(3.36 ,10.44 ) |
| Psychiatric disorders | Mood swings | 11 | 4.48（2.48 ,8.11 ） | 4.48（29.69 ） | 2.16(0.96 ,2.63 ) | + | 4.47(2.48 ,8.09 ) |
| General disorders and administration site conditions | Crying | 11 | 3.89（2.15 ,7.02 ） | 3.88（23.51 ） | 1.96(0.81 ,2.48 ) | + | 3.88(2.15 ,7.01 ) |
| Psychiatric disorders | Hallucination, visual | 11 | 4.68（2.59 ,8.45 ） | 4.67（31.68 ） | 2.22(1.00 ,2.67 ) | + | 4.66(2.58 ,8.43 ) |
| Gastrointestinal disorders | Swollen tongue | 11 | 4.18（2.31 ,7.56 ） | 4.18（26.53 ） | 2.06(0.89 ,2.56 ) | + | 4.17(2.31 ,7.54 ) |
| Psychiatric disorders | Euphoric mood | 11 | 12.18（6.73 ,22.03 ） | 12.16（112.24 ） | 3.6(1.82 ,3.49 ) | ++ | 12.12(6.70 ,21.92 ) |
| Psychiatric disorders | Fear | 11 | 5.95（3.29 ,10.76 ） | 5.94（45.16 ） | 2.57(1.24 ,2.91 ) | + | 5.93(3.28 ,10.73 ) |
| Investigations | Body temperature increased | 10 | 4.37（2.35 ,8.13 ） | 4.37（25.92 ） | 2.12(0.87 ,2.61 ) | + | 4.36(2.34 ,8.11 ) |
| Nervous system disorders | Presyncope | 10 | 4.14（2.23 ,7.70 ） | 4.14（23.74 ） | 2.05(0.81 ,2.56 ) | + | 4.13(2.22 ,7.68 ) |
| General disorders and administration site conditions | Thirst | 10 | 4.84（2.60 ,9.00 ） | 4.83（30.37 ） | 2.27(0.97 ,2.71 ) | + | 4.83(2.60 ,8.98 ) |
| Nervous system disorders | Brain fog | 10 | 6.74（3.62 ,12.54 ） | 6.73（48.71 ） | 2.75(1.27 ,3.02 ) | + | 6.72(3.61 ,12.50 ) |
| Psychiatric disorders | Abnormal behaviour | 10 | 4.24（2.28 ,7.88 ） | 4.23（24.66 ） | 2.08(0.84 ,2.58 ) | + | 4.23(2.27 ,7.86 ) |
| Psychiatric disorders | Tachyphrenia | 9 | 30.98（16.06 ,59.79 ） | 30.95（257.99 ） | 4.94(2.03 ,3.87 ) | ++ | 30.62(15.87 ,59.09 ) |
| Eye disorders | Eye movement disorder | 9 | 14.78（7.67 ,28.47 ） | 14.76（114.89 ） | 3.88(1.72 ,3.55 ) | ++ | 14.69(7.63 ,28.30 ) |
| Psychiatric disorders | Panic reaction | 9 | 13.07（6.79 ,25.16 ） | 13.05（99.70 ） | 3.7(1.65 ,3.48 ) | ++ | 13(6.75 ,25.03 ) |
| Psychiatric disorders | Enuresis | 9 | 22.78（11.82 ,43.91 ） | 22.75（185.68 ） | 4.5(1.92 ,3.75 ) | ++ | 22.58(11.71 ,43.53 ) |
| Psychiatric disorders | Catatonia | 9 | 16.99（8.82 ,32.73 ） | 16.97（134.46 ） | 4.08(1.79 ,3.62 ) | ++ | 16.87(8.76 ,32.51 ) |
| Psychiatric disorders | Apathy | 9 | 6.83（3.55 ,13.14 ） | 6.82（44.60 ） | 2.77(1.19 ,3.02 ) | + | 6.81(3.54 ,13.10 ) |
| Vascular disorders | Peripheral coldness | 8 | 5.69（2.84 ,11.39 ） | 5.69（30.85 ） | 2.51(0.94 ,2.86 ) | + | 5.68(2.84 ,11.37 ) |
| Reproductive system and breast disorders | Sexual dysfunction | 8 | 7.37（3.68 ,14.75 ） | 7.36（43.84 ） | 2.88(1.14 ,3.07 ) | + | 7.34(3.67 ,14.70 ) |
| Psychiatric disorders | Initial insomnia | 8 | 8.25（4.12 ,16.52 ） | 8.24（50.77 ） | 3.04(1.23 ,3.15 ) | + | 8.22(4.11 ,16.46 ) |
| Nervous system disorders | Dizziness postural | 8 | 6.97（3.48 ,13.95 ） | 6.96（40.75 ） | 2.8(1.10 ,3.03 ) | + | 6.95(3.47 ,13.91 ) |
| Psychiatric disorders | Abnormal dreams | 7 | 4.32（2.06 ,9.07 ） | 4.32（17.82 ） | 2.11(0.59 ,2.63 ) | + | 4.31(2.05 ,9.06 ) |
| Psychiatric disorders | Bruxism | 7 | 14.38（6.84 ,30.23 ） | 14.37（86.63 ） | 3.84(1.40 ,3.45 ) | + | 14.3(6.80 ,30.06 ) |
| Psychiatric disorders | Somnambulism | 7 | 14.58（6.94 ,30.66 ） | 14.57（88.02 ） | 3.86(1.41 ,3.45 ) | + | 14.5(6.90 ,30.48 ) |
| General disorders and administration site conditions | Feeling of body temperature change | 7 | 12.33（5.87 ,25.92 ） | 12.32（72.49 ） | 3.62(1.33 ,3.37 ) | + | 12.27(5.84 ,25.79 ) |
| Nervous system disorders | Coordination abnormal | 7 | 7.98（3.80 ,16.77 ） | 7.98（42.61 ） | 2.99(1.07 ,3.11 ) | + | 7.96(3.79 ,16.72 ) |
| Psychiatric disorders | Logorrhoea | 7 | 29.34（13.93 ,61.80 ） | 29.31（189.45 ） | 4.86(1.66 ,3.71 ) | ++ | 29.02(13.78 ,61.13 ) |
| Psychiatric disorders | Self-injurious ideation | 6 | 15.92（7.13 ,35.53 ） | 15.91（83.36 ） | 3.98(1.25 ,3.44 ) | + | 15.82(7.09 ,35.32 ) |
| Psychiatric disorders | Bipolar disorder | 6 | 6.27（2.81 ,13.97 ） | 6.26（26.49 ） | 2.64(0.74 ,2.93 ) | + | 6.25(2.81 ,13.94 ) |
| Renal and urinary disorders | Incontinence | 6 | 5.74（2.58 ,12.80 ） | 5.74（23.43 ） | 2.52(0.68 ,2.87 ) | + | 5.73(2.57 ,12.77 ) |
| Nervous system disorders | Sensory disturbance | 6 | 4.7（2.11 ,10.47 ） | 4.69（17.42 ） | 2.23(0.53 ,2.71 ) | + | 4.69(2.10 ,10.45 ) |
| Psychiatric disorders | Bradyphrenia | 6 | 7.92（3.55 ,17.65 ） | 7.91（36.14 ） | 2.98(0.90 ,3.08 ) | + | 7.89(3.54 ,17.60 ) |
| Psychiatric disorders | Autoscopy | 6 | 61.72（27.48 ,138.62 ） | 61.67（350.45 ） | 5.92(1.57 ,3.77 ) | ++ | 60.37(26.88 ,135.60 ) |
| Psychiatric disorders | Dysphemia | 6 | 14.63（6.56 ,32.64 ） | 14.62（75.74 ） | 3.86(1.21 ,3.40 ) | + | 14.55(6.52 ,32.46 ) |
| Nervous system disorders | Formication | 6 | 11.31（5.07 ,25.23 ） | 11.31（56.14 ） | 3.49(1.10 ,3.28 ) | + | 11.26(5.05 ,25.12 ) |
| Skin and subcutaneous tissue disorders | Skin warm | 5 | 8.89（3.70 ,21.40 ） | 8.89（34.90 ） | 3.15(0.76 ,3.12 ) | + | 8.86(3.68 ,21.33 ) |
| Nervous system disorders | Slow speech | 5 | 17.18（7.13 ,41.40 ） | 17.17（75.69 ） | 4.09(1.03 ,3.40 ) | + | 17.07(7.09 ,41.14 ) |
| Psychiatric disorders | Negative thoughts | 5 | 19.95（8.27 ,48.08 ） | 19.93（89.29 ） | 4.31(1.08 ,3.44 ) | + | 19.8(8.21 ,47.73 ) |
| Gastrointestinal disorders | Tongue movement disturbance | 5 | 40.64（16.80 ,98.28 ） | 40.61（190.43 ） | 5.32(1.23 ,3.60 ) | + | 40.05(16.56 ,96.85 ) |
| Nervous system disorders | Drooling | 5 | 7.01（2.92 ,16.88 ） | 7.01（25.70 ） | 2.81(0.63 ,2.99 ) | + | 7(2.91 ,16.83 ) |
| Injury, poisoning and procedural complications | Sedation complication | 5 | 11.31（4.70 ,27.24 ） | 11.31（46.79 ） | 3.49(0.87 ,3.24 ) | + | 11.26(4.68 ,27.12 ) |
| General disorders and administration site conditions | Screaming | 5 | 10.52（4.37 ,25.33 ） | 10.51（42.89 ） | 3.39(0.84 ,3.20 ) | + | 10.48(4.35 ,25.23 ) |
| Musculoskeletal and connective tissue disorders | Posture abnormal | 4 | 12.84（4.81 ,34.29 ） | 12.83（43.45 ） | 3.68(0.63 ,3.22 ) | + | 12.78(4.78 ,34.13 ) |
| Psychiatric disorders | Distractibility | 4 | 32.6（12.16 ,87.37 ） | 32.58（121.05 ） | 5.01(0.85 ,3.45 ) | + | 32.22(12.02 ,86.36 ) |
| Investigations | Drug screen positive | 4 | 12.81（4.80 ,34.22 ） | 12.8（43.33 ） | 3.67(0.63 ,3.22 ) | + | 12.75(4.77 ,34.06 ) |
| Psychiatric disorders | Tic | 4 | 8.99（3.37 ,24.00 ） | 8.99（28.30 ） | 3.16(0.50 ,3.08 ) | + | 8.96(3.36 ,23.92 ) |
| Investigations | Body temperature fluctuation | 4 | 11.89（4.45 ,31.77 ） | 11.89（39.72 ） | 3.57(0.61 ,3.20 ) | + | 11.84(4.43 ,31.63 ) |
| Psychiatric disorders | Depressive symptom | 4 | 10.54（3.95 ,28.15 ） | 10.54（34.40 ） | 3.39(0.56 ,3.15 ) | + | 10.5(3.93 ,28.04 ) |
| Psychiatric disorders | Schizoaffective disorder bipolar type | 4 | 109.82（40.44 ,298.27 ） | 109.76（414.97 ） | 6.72(0.95 ,3.59 ) | + | 105.7(38.92 ,287.06 ) |
| General disorders and administration site conditions | Energy increased | 4 | 6.85（2.57 ,18.28 ） | 6.85（19.93 ） | 2.77(0.36 ,2.95 ) | + | 6.83(2.56 ,18.23 ) |
| Psychiatric disorders | Manic symptom | 4 | 174.03（63.39 ,477.80 ） | 173.93（647.88 ） | 7.36(0.95 ,3.62 ) | + | 163.91(59.70 ,450.01 ) |
| Nervous system disorders | Sleep paralysis | 4 | 24.7（9.23 ,66.11 ） | 24.68（90.12 ） | 4.61(0.81 ,3.40 ) | + | 24.48(9.15 ,65.52 ) |
| Psychiatric disorders | Soliloquy | 4 | 43.84（16.33 ,117.74 ） | 43.82（164.82 ） | 5.43(0.89 ,3.50 ) | + | 43.17(16.07 ,115.92 ) |
| Gastrointestinal disorders | Vomiting projectile | 4 | 15.12（5.66 ,40.41 ） | 15.11（52.44 ） | 3.91(0.69 ,3.28 ) | + | 15.04(5.63 ,40.19 ) |
| Social circumstances | Refusal of treatment by patient | 3 | 6.45（2.08 ,20.03 ） | 6.45（13.78 ） | 2.69(0.00 ,2.89 ) | + | 6.44(2.07 ,19.98 ) |
| Psychiatric disorders | Emotional poverty | 3 | 12.51（4.02 ,38.90 ） | 12.51（31.62 ） | 3.64(0.24 ,3.14 ) | + | 12.46(4.01 ,38.73 ) |
| Psychiatric disorders | Intrusive thoughts | 3 | 16.93（5.44 ,52.69 ） | 16.92（44.68 ） | 4.07(0.31 ,3.21 ) | + | 16.83(5.41 ,52.37 ) |
| Psychiatric disorders | Psychotic symptom | 3 | 9.39（3.02 ,29.19 ） | 9.39（22.42 ） | 3.23(0.15 ,3.04 ) | + | 9.36(3.01 ,29.09 ) |
| Psychiatric disorders | Dissociative disorder | 3 | 35.05（11.22 ,109.47 ） | 35.04（97.99 ） | 5.11(0.42 ,3.33 ) | + | 34.62(11.09 ,108.13 ) |
| Psychiatric disorders | Trance | 3 | 117.81（37.11 ,374.01 ） | 117.77（333.43 ） | 6.82(0.48 ,3.44 ) | + | 113.1(35.63 ,359.03 ) |
| Musculoskeletal and connective tissue disorders | Muscle fatigue | 3 | 6.77（2.18 ,21.04 ） | 6.77（14.73 ） | 2.76(0.02 ,2.92 ) | + | 6.76(2.18 ,20.99 ) |
| Psychiatric disorders | Homicidal ideation | 3 | 16.31（5.24 ,50.76 ） | 16.31（42.86 ） | 4.02(0.31 ,3.20 ) | + | 16.22(5.21 ,50.46 ) |
| Nervous system disorders | Pseudostroke | 3 | 69.53（22.11 ,218.64 ） | 69.5（197.69 ） | 6.08(0.47 ,3.40 ) | + | 67.86(21.58 ,213.38 ) |
| Nervous system disorders | Incoherent | 3 | 6.47（2.09 ,20.11 ） | 6.47（13.85 ） | 2.69(0.00 ,2.89 ) | + | 6.46(2.08 ,20.06 ) |
| Investigations | Blood prolactin increased | 3 | 6.74（2.17 ,20.94 ） | 6.74（14.63 ） | 2.75(0.02 ,2.91 ) | + | 6.73(2.17 ,20.89 ) |
| Psychiatric disorders | Anorgasmia | 3 | 13.34（4.29 ,41.47 ） | 13.33（34.06 ） | 3.73(0.26 ,3.15 ) | + | 13.27(4.27 ,41.28 ) |
| Psychiatric disorders | Flat affect | 3 | 22.15（7.11 ,68.99 ） | 22.14（60.08 ） | 4.46(0.36 ,3.27 ) | + | 21.97(7.05 ,68.45 ) |

Abbreviation: n, Number of cases reporting PT; ROR, Reporting odds ratio; CI, confdence interval; IC, information component; IC 025, the lower 95% CI of IC
